# Neuronal Mitophagy: Friend or Foe?

**DOI:** 10.3389/fcell.2020.611938

**Published:** 2021-01-18

**Authors:** Christina Doxaki, Konstantinos Palikaras

**Affiliations:** ^1^General University Hospital of Heraklion, Crete, Greece; ^2^Institute of Molecular Biology and Biotechnology, Foundation for Research and Technology-Hellas, Crete, Greece

**Keywords:** aging, cell death, energy metabolism, homeostasis, mitochondria, mitophagy, neurodegeneration, neuroprotection

## Abstract

Maintenance of neuronal homeostasis is a challenging task, due to unique cellular organization and bioenergetic demands of post-mitotic neurons. It is increasingly appreciated that impairment of mitochondrial homeostasis represents an early sign of neuronal dysfunction that is common in both age-related neurodegenerative as well as in neurodevelopmental disorders. Mitochondrial selective autophagy, known as mitophagy, regulates mitochondrial number ensuring cellular adaptation in response to several intracellular and environmental stimuli. Mounting evidence underlines that deregulation of mitophagy levels has an instructive role in the process of neurodegeneration. Although mitophagy induction mediates the elimination of damaged mitochondria and confers neuroprotection, uncontrolled runaway mitophagy could reduce mitochondrial content overstressing the remaining organelles and eventually triggering neuronal cell death. Unveiling the molecular mechanisms of neuronal mitophagy and its intricate role in neuronal survival and cell death, will assist in the development of novel mitophagy modulators to promote cellular and organismal homeostasis in health and disease.

## Introduction

Mitochondria are remarkably dynamic organelles that divide, fuse and migrate in different cellular compartments. The processes of mitochondrial fission and fusion ensure metabolite and mitochondrial DNA (mtDNA) exchange for dilution of dysfunctional elements as well as dictate organelle shape, number and bioenergetic functionality. Neurons require high energy levels and depend on mitochondrial homeostasis to carry out their functions and sustain neuronal circuit formation, communication and activity (Misgeld and Schwarz, 2017; Palikaras and Tavernarakis, 2020). Hence, neuronal cells are equipped with specialized molecular mechanisms for efficient distribution of mitochondria. Mitochondria are typically localized to areas of high-energy demand including the distal portion of the initial segment of axons, the nodes of Ranvier, growth cones, presynaptic buttons and postsynaptic densities (Steketee et al., 2012; Smith et al., 2016; Misgeld and Schwarz, 2017; Garcia et al., 2019; Verreet et al., 2019). While progress has been made in identifying the proteins involved in mitochondrial transport within neurons, the significance of localizing mitochondria in neuronal compartments, where they can respond to local changes in neuronal activity and energy metabolism, is largely unknown and only starting to be exploited (Sheng and Cai, 2012; Misgeld and Schwarz, 2017; Garcia et al., 2019; Palikaras and Tavernarakis, 2020).

It becomes increasingly appreciated that the removal of misfolded proteins, protein aggregates and damaged mitochondria, is of crucial importance for the proper function and long-term survival of neurons. The detailed molecular mechanisms that govern mitochondrial selective autophagy in such a highly differentiated and compartmentalized cell, as well as their relevance to neuronal physiology only now begin to be elucidated. During the last decade, mitophagy was widely studied in the context of stress and pathological conditions (Palikaras et al., 2018; Lou et al., 2020; Yan et al., 2020). A growing body of evidence highlights mitophagy as a physiological process that occurs constitutively at baseline levels in the nervous system. Interestingly, basal mitophagy levels differ between brain regions and neuronal sub-populations. The dentate gyrus, lateral ventricle and Purkinje cells display high levels of mitophagy, whereas mitophagy is low in the neurons of the striatum, cortex and substantia nigra (Sun et al., 2015). Furthermore, neuronal mitophagy declines with age leading to accumulation of defective organelles (Palikaras et al., 2015; Sun et al., 2015; Evans and Holzbaur, 2020a). Increased mitochondrial damage is a hallmark of aging and age-associated neurodegeneration highlighting that neuronal cells are particularly sensitive to age-dependent mitophagy impairment.

While the role of mitophagy in cellular and organismal physiology is essential, several pathological conditions, such as mitochondrial disorders, ischemic stroke, chronic cerebral hypoperfusion, and diabetes, are shown to stimulate uncontrolled mitochondrial elimination that subsequently leads to neuronal cell death (Shi et al., 2014; Su et al., 2018; Devi et al., 2019; Park et al., 2019; Zaninello et al., 2020). These findings underline mitophagy as a “double-edged sword” for neuronal homeostasis and viability. Here, we survey recent advances toward the elucidation of the intricate role of mitophagy in neuronal survival and cell death.

## Molecular Mechanisms of Mitophagy in Neuronal Cells

**Figure 1:**
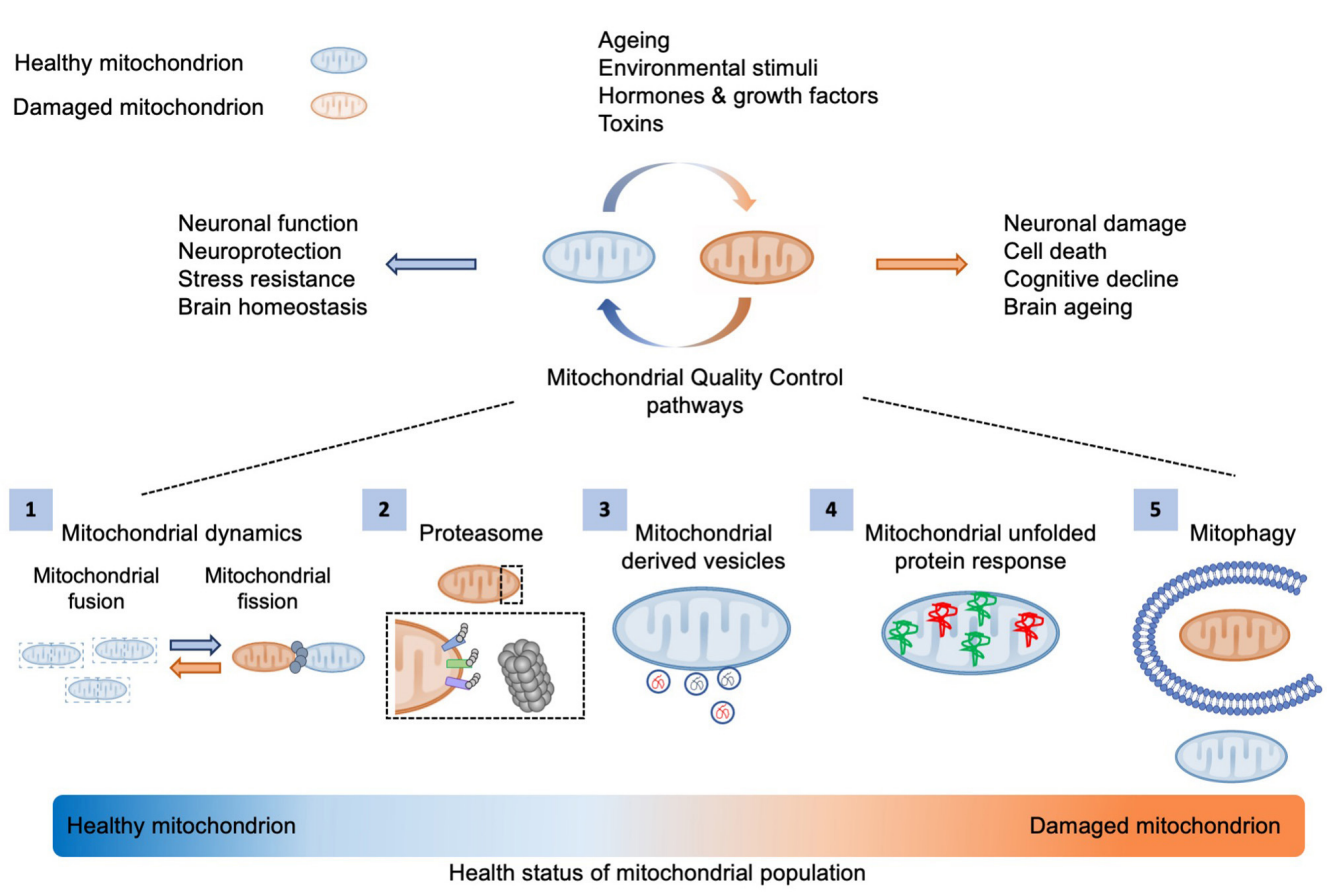
Mitochondrial quality control mechanisms. Several environmental and intracellular stimuli influence mitochondrial activity and integrity. The maintenance of a healthy mitochondrial population is essential for cellular metabolism and brain function. Thus, aged or defective organelles need either to be restored via mitochondrial quality control mechanisms, including (1) mitochondrial dynamics, (2) proteasome system, (3) mitochondrial derived vesicles (MDVs), and (4) mitochondrial unfolded protein response (UPRmt), or (5) be degraded by mitophagy.

The maintenance of a healthy mitochondrial pool is pivotal for cellular and organismal homeostasis (Figure 1). Post-mitotic neuronal cells are more susceptible to mitochondrial damage due to their increased energetic demands. Therefore, aged or dysfunctional mitochondria need either to be repaired through mitochondrial surveillance quality mechanisms, including proteasome system, mitochondrial proteases, mitochondrial derived vesicles (MDVs), fission-fusion machinery and mitochondrial unfolded protein response (UPRmt), or be eliminated by selective mitochondrial autophagy (Figure 1) (Palikaras et al., 2018).

Several molecular mechanisms have been uncovered to mediate mitochondrial removal to date, highlighting that mitophagy could be triggered in response to diverse stimuli via multiple signaling pathways, in distinct cellular contexts. Accumulating evidence demonstrates an intricate coordination between mitophagy regulatory mechanisms and highlights their conservation from yeast to mammals (Palikaras et al., 2018; Pickles et al., 2018; Lou et al., 2020). The PINK1 (PTEN-induced putative kinase 1)/Parkin pathway is the most well-characterized signaling cascade that orchestrates mitochondrial degradation in response to stress (Harper et al., 2018; Palikaras et al., 2018; Montava-Garriga and Ganley, 2020). PINK1 is a mitochondrial protein kinase that is stabilized on the outer mitochondrial membrane upon challenged conditions. PINK1 activity is induced upon its auto-phosphorylation leading to the recruitment of Parkin on mitochondrial surface (Hasson et al., 2013; Lazarou et al., 2015; Khaminets et al., 2016; Pickles et al., 2018; Sekine and Youle, 2018). In turn, Parkin ubiquitinates various outer mitochondrial membrane proteins promoting either their degradation or their association with autophagy adaptors and general autophagic machinery (Hasson et al., 2013; Heo et al., 2015; Lazarou et al., 2015; Khaminets et al., 2016; Gatica et al., 2018; Pickles et al., 2018; Sekine and Youle, 2018). Several studies underlie the causative interplay between PINK1 and Parkin activation in response to mitochondrial dysfunction. Systematic proteomic analysis both in non-neuronal and neuronal cells suggest the stimulation of a feed-forward mechanism that involves the PINK1-dependent phosphorylation of ubiquitin (Ub) and poly-Ub chains on damaged organelles to enhance mitophagy signal upon stress (Harper et al., 2018; Ordureau et al., 2018, 2020). Furthermore, several PINK1- and Parkin-independent molecular pathways have been identified implicating the significance of multiple mitochondrial proteins or lipids, including FUNDC1 (FUN14 Domain Containing 1), BNIP3 (BCL2/adenovirus E1B 19-kDa-interacting protein 3), NIX/BNIP3L (BCL2/adenovirus E1B 19-kDa-interacting protein 3-Like), BCL2L13 (BCL2- Like 13), FKBP8 (FK506 binding protein 8), PHB2, cardiolipin, and ceramide among others, which act as receptors and facilitate mitophagy (Sentelle et al., 2012; Harper et al., 2018; Palikaras et al., 2018; Montava-Garriga and Ganley, 2020).

The removal of neuronal mitochondria is a challenging cellular event since the majority of mitochondrial population is located at the distal neuronal compartments, far away from the cell body of the neuron, where mature acidic lysosomes mainly present (Holtzman and Novikoff, 1965; Cai et al., 2010; Evans and Holzbaur, 2020b; Han et al., 2020). Despite the spatial limitations, timely degradation of impaired mitochondria is essential for neuronal protection against cell death. Recent studies have suggested that Parkin-mediated mitophagy is limited in a small subset of mature neurons and takes place much more slowly than in other cell types (Cai et al., 2012a,b; Lin et al., 2017; Puri et al., 2019). These results support the notion that alternative molecular mechanisms sustain mitochondrial homeostasis upon mild stress, before the stimulation of Parkin-mediated mitophagy. Indeed, a very recent study has demonstrated that the mitochondrial E3 ubiquitin ligase 1 (Mul1) facilitates an early checkpoint to preserve mitochondrial integrity and restrains neuronal mitophagy under mild mitochondrial stress (Puri et al., 2019). Interestingly, Mul1 affects the stability of Mitofusin 2 (MFN2) preserving mitochondrial homeostasis. Mul1 deficiency increases MFN2 protein levels promoting hyperfusion of the mitochondrial network. Notably, MFN2 is also located on the endoplasmic reticulum (ER)-mitochondrial contact sites where it regulates calcium homeostasis (De Brito and Scorrano, 2008; Filadi et al., 2015; Mclelland et al., 2018). Therefore, depletion of Mul1 in neurons results in impaired ER-mitochondrial tethering due to MFN2 upregulation, which in turn triggers the elevation of cytoplasmic calcium levels, calcineurin activation, DRP1 (Dynamin-related protein 1)-mediated mitochondrial fragmentation and eventually mitophagy (Puri et al., 2019). Studies in non-neuronal cells have shown that MFN2 is phosphorylated by PINK1, and thereby promotes Parkin recruitment on the outer mitochondrial membrane (Chen and Dorn, 2013). In turn, Parkin ubiquitinates MFN2 to induce its degradation, which results in the subsequent release of mitochondria from the ER, thereby enhancing mitophagy (Chen and Dorn, 2013; Mclelland et al., 2018). Although the core mitophagy constituents are conserved in both neurons and non-neuronal cells, the kinetics of mitochondrial elimination varies not only between different cell types, but also between neuronal sub-populations. These results indicate the existence of a multi-step, highly organized and precise mitochondrial quality control system.

A recent study reported that the degradation of defective organelles is a rate-limiting event in neuronal cells (Evans and Holzbaur, 2020a). Mild stress conditions trigger mitochondrial depolarization, resulting in their subsequent sequestration by autophagosomes and their delivery from the distal neuronal compartments to the soma. Although mitophagy in non-neuronal cells is taking place rapidly, neuronal mitoautophagosomes remain intact in non-acidified organelles, whereby their elimination is a very slow process (Evans and Holzbaur, 2020a). Optineurin (OPTN) and TANK-binding kinase 1 (TBK1) act downstream of Parkin to recognize defective organelles and promote their autophagosomal engulfment and retrograde transportation under antioxidant deprivation. Interestingly, OPTN-mediated mitophagy is spatially-restricted in neuronal cell bodies, with only few mitophagic events to be detected in axons or dendrites (Evans and Holzbaur, 2020a). These results further support the notion that mitophagy is differentially regulated across neuronal compartments, as it is previously demonstrated both *in vitro* and *in vivo* (Ashrafi et al., 2014; Devireddy et al., 2015; Mcwilliams et al., 2016; Sung et al., 2016; Puri et al., 2019; Zaninello et al., 2020). Despite the fact that several adaptor molecules, such as NDP52, OPTN, p62 and TAX1BP1 (Tax1 binding protein 1), have been identified to facilitate mitophagy in non-neuronal cells, it remains still elusive whether these adaptor proteins might participate and/or co-regulate the highly compartmentalized nature of neuronal mitophagy.

The differential kinetic patterns of mitochondrial turnover under both basal and challenged conditions could explain the enhanced vulnerability of neurons upon mitochondrial dysfunction. Additionally, age-dependent perturbations in cellular homeostasis, including proteostasis collapse, lysosomal dysfunction, mitochondrial impairment, increased oxidative stress and genomic instability among others, could further impede the efficiency of mitophagy exacerbating neuronal susceptibility to degeneration (Hou et al., 2018; Palikaras et al., 2018; Hipp et al., 2019; Lie and Nixon, 2019).

## Neuroprotective Role of Mitophagy

**Figure 2:**
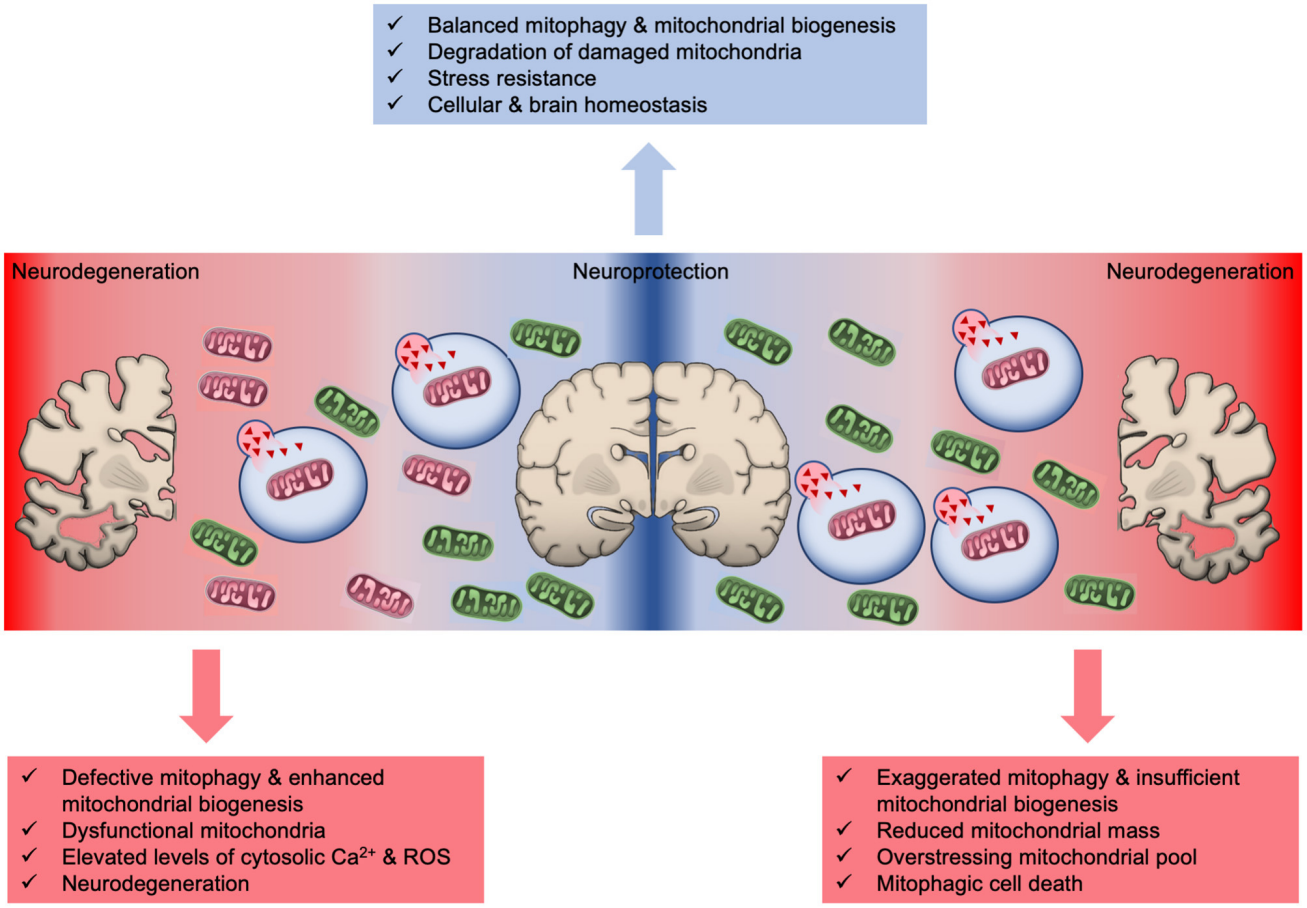
The intricate role of mitophagy in neuroprotection and neurodegeneration. Basal mitophagy eliminates defective mitochondria and simultaneously new organelles are generated through mitochondrial biogenesis to sustain mitochondrial population. Excessive mitophagy significantly reduces mitochondrial number overstressing the remaining organelles and eventually leads to energetic crisis and cell death. Similarly, mitophagy defects results in the accrual of damaged mitochondria promoting neurodegeneration and brain aging.

Defective mitochondrial turnover results in the progressive accumulation of damaged organelles and is characterized as a hallmark of aging and age-related neurodegenerative pathologies (Figure 2) (Hou et al., 2019; Lautrup et al., 2019; Cai and Jeong, 2020; Lou et al., 2020). Post-mortem Alzheimer's disease (AD) hippocampal samples display smaller mitochondria with altered cristae formation and impaired function compared with their counterparts in age-matched healthy controls (Fang et al., 2019). Further evidence stemming from human induced pluripotent stem cells (iPSCs)-derived AD neurons indicated significantly decreased phosphorylation levels of ULK1 (Unc-51 Like Autophagy Activating Kinase 1) and TBK1, thereby suggesting that the initiation of the mitophagy process is impaired (Fang et al., 2019). Congruently, levels of other essential autophagy/mitophagy regulators, such as Parkin, OPTN, Mul1, BECN1 (Beclin 1), AMBRA1 (Autophagy And Beclin 1 Regulator 1), FUNDC1, are diminished in AD samples (Pickford et al., 2008; Martin-Maestro et al., 2016; Fang et al., 2019). Moreover, a recent study demonstrated that overexpression of wild type and disease-associated tau results in reduced Parkin recruitment onto mitochondrial surface and mitophagy inhibition in both nematodes and neuroblastoma cells (Cummins et al., 2019). These findings suggest that the impairment of basal mitophagy is an early event in AD human brain and a causative factor in the development and progression of disease pathophysiology (Kerr et al., 2017; Fang et al., 2019).

In addition to AD, mitochondrial abnormalities and mitophagy defects have also been associated with the pathogenesis of Parkinson's disease (PD) (Liu et al., 2019). Loss of function mutations of *PARK6* and *PARK2* genes, which encode PINK1 and Parkin respectively, are linked to familial form of parkinsonism (Chu, 2019). Although the PINK1/Parkin pathway has been extensively studied in both neuronal and non-neuronal cell cultures, its role in mitophagy regulation and neuronal survival *in vivo* remains still controversial. Studies in flies and rats demonstrated that Parkin deficiency leads to altered mitochondrial morphology and function accompanied by dopaminergic neuronal loss and motility defects (Kitada et al., 1998; Whitworth et al., 2005; Yang et al., 2006; Dave et al., 2014; Pickrell and Youle, 2015). However, PINK1 and Parkin deficiency does not trigger any severe PD-associated phenotype in mice, suggesting the existence of compensatory mitochondrial quality control mechanisms (Goldberg et al., 2003; Perez and Palmiter, 2005). Further supporting this notion, transgenic flies and mice expressing mitophagy reporter identified that PINK1 and Parkin are dispensable for basal neuronal mitophagy (Lee et al., 2018; Mcwilliams et al., 2018). Interestingly, a recent study in mice identified that exhaustive exercise and increased mtDNA damage stimulate dopaminergic neuronal loss and locomotion defects in both PINK1 and Parkin mutant mice (Sliter et al., 2018). These findings underscore that acute and/or chronic organismal stress might contribute to the development and progression of diverse pathological conditions, especially in sensitive genetic backgrounds.

Mounting evidence suggests that mutant huntingtin (mHtt) influences directly mitochondrial homeostasis contributing to Huntington's disease (HD) pathogenesis (Orr et al., 2008; Shirendeb et al., 2011; Yan et al., 2020). Furthermore, autophagy-mediated degradation of both cytoplasmic and mitochondrial constituents is perturbed in HD leading to the progressive accumulation of protein aggregates and dysfunctional mitochondria (Martinez-Vicente et al., 2010). Indeed, microscopic examination of the dentate gyrus region revealed that basal mitophagy levels are diminished in a mouse model of HD compared to age-matched control animals (Sun et al., 2015). Supporting the neuroprotective role of mitophagy, genetic studies in flies and rodents uncovered that mHtt is associated directly with mitochondrial membranes and impairs mitochondrial integrity and mitophagy execution leading to neurodegeneration (Hwang et al., 2015; Khalil et al., 2015; Guo et al., 2016; Franco-Iborra et al., 2020).

Pharmacological upregulation of mitophagy is shown to promote neuroprotection against several neurodegenerative pathologies (Georgakopoulos et al., 2017; Palikaras et al., 2018). Indeed, dietary supplementation with urolithin A (UA), actinonin and NAD^+^ (Nicotinamide Adenine Dinucleotide)-precursor molecules, restores multiple pathological features of AD, including mitochondrial damage, neuronal defects, Aβ aggregation, aberrant tau phosphorylation levels, neuroinflammation, and cognitive dysfunction among others (Hou et al., 2018; Fang, 2019; Lautrup et al., 2019; Gilmour et al., 2020). Moreover, nicotinamide riboside (NR; NAD^+^-precursor molecules) administration preserves mitochondrial function and diminishes degeneration of dopaminergic neurons through mitophagy induction in flies, mouse and iPSCs models of PD (Schondorf et al., 2018). Recent clinical trials suggest that administration of both NR and UA is safe without demonstrating any adverse effect in compound-treated compared to placebo-treated group and highlight their therapeutic potential (Dollerup et al., 2018; Andreux et al., 2019; Conze et al., 2019; Radenkovic et al., 2020). Interestingly, NAD^+^-precursor molecules confer neuroprotection through the activation of BNIP3-mediated mitophagy in both AD and PD models (Schondorf et al., 2018; Fang et al., 2019). These results underline the requirement of personalized pharmacological approaches that depend on the patient genetic background to overcome mitophagy pathway deficiencies in pathological conditions (Xie et al., 2019).

Spermidine, a natural occurring compound, is shown to induce autophagy and mitophagy promoting cytoprotection, stress resistance and longevity in yeast cells, nematodes, flies, and mice (Eisenberg et al., 2009, 2016; Madeo et al., 2018). Furthermore, spermidine-rich feeding reverses the age-induced decline of polyamines in brain tissue and subsequently improves synaptic impairment and memory loss in an autophagy-dependent manner (Gupta et al., 2013, 2016; Maglione et al., 2019). A randomized clinical trial was recently conducted to evaluate the impact of spermidine supplementation on memory performance in humans (Wirth et al., 2018). Spermidine-treated aged-individuals displayed moderate improvement in the memory performance and enhanced mnemonic discrimination abilities compared to placebo-treated group (Wirth et al., 2018). The decreased levels of spermidine in blood serum of AD patients compared to healthy individuals, further support the therapeutic potential of polyamine-rich food supplementation against age-related dementia in humans (Madeo et al., 2018; Joaquim et al., 2019).

Taken together, the central role of mitophagy in neuronal communication, activity and survival is steadily emerging. It is increasingly appreciated that impairment of mitochondrial metabolism represents an early sign of neuronal deficits that is common in both age-related deterioration of brain function as well as in neurodegenerative disorders. Therefore, the development of novel therapeutic interventions to modulate mitochondrial turnover and preserve healthy mitochondrial population could confer neuroprotection against brain aging before any irreversible impairment.

## Mitophagic Cell Death in Neurons

The tight coordination between mitochondrial biogenesis and degradation is essential for cellular, tissue and organismal physiology (Palikaras and Tavernarakis, 2014; Palikaras et al., 2015). Insufficient mitochondrial biogenesis and excessive mitophagy diminish mitochondrial population that burdens the remaining organelles, which subsequently promote mitophagy-mediated cell death (Figure 2) (Palikaras and Tavernarakis, 2014; Subramaniam, 2020). Indeed, excessive mitochondrial elimination has been indicated as a cause of cell death in several disease models (Subramaniam, 2020). Notably, BNIP3-mediated mitophagy is shown to promote neuronal death both *in vitro* and *in vivo* upon ischemic stroke (Shi et al., 2014). BNIP3 deficiency prevents neuronal loss in neonatal brains upon ischemia/hypoxia (I/H) treatment. Although BNIP3 and NIX/BNIP3L share high degree of sequence homology, elevated NIX/BNIP3L levels could not compensate BNIP3 depletion to trigger mitophagy and cell death in response to ischemic stroke. Therefore, it is suggested that mitophagic cell death is mainly regulated by BNIP3, whereas NIX/BNIP3L sustains basal mitophagy levels in neurons (Shi et al., 2014). Further supporting the pro-death activities of BNIP3, a recent study demonstrated increased protein levels of BNIP3 and mitophagy upregulation in a rat model of chronic cerebral hyperfusion (CCH). Chronic state of reduced cerebral blood flow leads to CCH that is linked to neurological damage and cognitive decline (Somredngan and Thong-Asa, 2018). Interestingly, administration of URB597, a fatty acid amide hydrolase inhibitor, decreases BNIP3 and Parkin protein levels, which confers neuroprotection due to mitophagy inhibition (Somredngan and Thong-Asa, 2018).

The brain region hippocampus retains its neurogenic capacities throughout adulthood and is severely affected during brain aging or injury. Evidently, neuronal stem cell plasticity, survival and differentiation are under constant and tight proteostatic and metabolic regulation (Garcia-Prat et al., 2017; Khacho et al., 2019). Moreover, transgenic mice expressing mitophagy reporter display increased basal mitophagy levels in the hippocampal dentate gyrus, highlighting the pivotal role of mitochondrial quality control in neurogenesis (Sun et al., 2015; Khacho et al., 2019). Hippocampal neuronal stem cells (HNSCs) differentiation and viability depends on extracellular stimuli, including insulin/insulin-like growth factors (IGFs). It is already documented that insulin depletion triggers autophagy-dependent cell death in HNSCs (Aberg et al., 2000; Lichtenwalner et al., 2001). Parkin stimulation deregulates ER-mitochondrial tethering promoting cytoplasmic calcium elevation and mitophagic cell death in hippocampal neurons upon insulin withdrawal (Park et al., 2019). Altered insulin signaling interferes with hippocampal neuronal function and is associated with several neurodegenerative and psychiatric disorders (Bernstein et al., 2020; Palikaras and Tavernarakis, 2020). Taken together these results underline the impact of imbalanced mitochondrial biogenesis and mitophagy in neuronal stem cell survival.

Recent findings demonstrated that excessive mitophagy eliminates mitochondrial content in striatal neurons and triggers cell death upon mitochondrial damage (Sharma et al., 2019). The small GTPase Rhes (Ras homolog enriched in striatum) is highly expressed in striatum brain region and is associated with lysosomal and mitochondrial cellular compartments. Interestingly, Rhes induces NIX-mediated runaway mitophagy and reduces mitochondrial content in response to 3-Nitropropionic acid (3-NP) mitochondrial toxicant (Sharma et al., 2019). Evidently, 3-NP supplementation promotes striatal lesions and causes severe pathological symptoms in mammals reminiscent of HD (Brouillet et al., 1993; Fu et al., 1995; He et al., 1995; Guyot et al., 1997). Therefore, it is tempting to speculate that imbalance of mitophagy might be a contributing factor to striatal neurodegeneration and pathogenesis of HD.

Several studies in mammals and nematodes demonstrated that disruption of the continual redistribution of mitochondria compromises axonal stability, synaptic integrity and neuropeptide release leading to neurodegeneration (Misko et al., 2012; Cherra et al., 2013; Rawson et al., 2014; Zhao et al., 2018; Han et al., 2020; Zaninello et al., 2020). A very recent study reported that exaggerated autophagy/mitophagy depletes axonal mitochondria in retinal ganglion cells (RGCs) and mediates the development of autosomal optic atrophy (ADOA) (Zaninello et al., 2020). ADOA is an incurable genetic disorder caused by mutations in the *OPA1* gene (Alexander et al., 2000; Delettre et al., 2000). Although, the main pathological feature of ADOA is optic nerve degeneration and visual loss during early childhood, several patients develop multi-systemic deteriorations, including deafness, ataxia, myopathies and paraplegia among others, underlining the pivotal role of OPA1 activity in the maintenance of mitochondrial metabolism and tissue homeostasis (Yu-Wai-Man et al., 2010; Belenguer and Pellegrini, 2013). Further elaborating on the contribution of mitophagy to ADOA pathogenesis, it is documented that OPA1 mutation stimulates AMPK (AMP-activated protein kinase) activity in axonal hillock and mediates mitochondrial degradation in nematode and mouse neurons. Notably, autophagy deficiency restores mitochondrial content in axons and inhibits visual defects in an ADOA mouse model highlighting the detrimental effect of uncontrolled mitophagy in disease development and progression (Zaninello et al., 2020).

## Conclusion

Mitophagy preserves energy homeostasis facilitating the elimination of defective organelles and, thereby, preventing the release of various harmful byproducts of mitochondrial activity. Hence, mitophagy stimulation exerts cytoprotection against premature aging and neuronal death (Palikaras et al., 2018; Bakula and Scheibye-Knudsen, 2020). Although the molecular mechanisms of mitochondrial selective autophagy have been extensively studied, several controversial questions remain to be addressed about neuronal mitophagy. The stimulus and the molecular mechanisms, which regulate autophagosomal formation in axons, cargo recognition and sequestration, transport to the soma, fusion with lysosomes and degradation are still obscure. Furthermore, several gaps remain in our understanding of major aspects of mitochondrial contributions to neuronal vulnerability and aging that might trigger cellular and/or tissue damage.

Aging represents the greatest risk factor for the onset of degenerative diseases of the nervous system. These include the very common Alzheimer's, Parkinson's, and Huntington's diseases among others, all of which are tightly associated with mitochondrial and autophagic defects (Chu, 2019; Bakula and Scheibye-Knudsen, 2020; Cai and Jeong, 2020; Evans and Holzbaur, 2020b; Lou et al., 2020; Yan et al., 2020). Additionally, the molecular pathways that orchestrate the crosstalk between mitophagy, apoptosis and necrotic cell death in axonal and dendritic degeneration remain unknown. Unraveling potent chemical modulators of mitophagy and understanding how mitochondrial elimination promotes either neuroprotection or neuronal cell death would be essential for the development of novel and context-specific pharmacological interventions against neurodegenerative disorders.

## Author Contributions

CD and KP wrote and edited the manuscript. KP supervised manuscript preparation. All authors contributed to the article and approved the submitted version.

## Conflict of Interest

The authors declare that the research was conducted in the absence of any commercial or financial relationships that could be construed as a potential conflict of interest.

## References

[B1] Aberg M. A. Aberg N. D. Hedbacker H. Oscarsson J. Eriksson P. S. (2000). Peripheral infusion of IGF-I selectively induces neurogenesis in the adult rat hippocampus. J. Neurosci. 20, 2896–2903. 10.1523/JNEUROSCI.20-08-02896.2000 10751442 PMC6772218

[B2] Alexander C. Votruba M. Pesch U. E. Thiselton D. L. Mayer S. Moore A.. (2000). OPA1, encoding a dynamin-related GTPase, is mutated in autosomal dominant optic atrophy linked to chromosome 3q28. Nat. Genet. 26, 211–215. 10.1038/79944 11017080

[B3] Andreux P. A. Blanco-Bose W. Ryu D. Burdet F. Ibberson M. Aebischer P.. (2019). The mitophagy activator urolithin A is safe and induces a molecular signature of improved mitochondrial and cellular health in humans. Nat. Metab. 1, 595–603. 10.1038/s42255-019-0073-4 32694802

[B4] Ashrafi G. Schlehe J. S. Lavoie M. J. Schwarz T. L. (2014). Mitophagy of damaged mitochondria occurs locally in distal neuronal axons and requires PINK1 and Parkin. J. Cell Biol. 206, 655–670. 10.1083/jcb.201401070 25154397 PMC4151150

[B5] Bakula D. Scheibye-Knudsen M. (2020). MitophAging: mitophagy in aging and disease. Front. Cell Dev. Biol. 8:239. 10.3389/fcell.2020.00239 32373609 PMC7179682

[B6] Belenguer P. Pellegrini L. (2013). The dynamin GTPase OPA1: more than mitochondria? Biochim. Biophys. Acta 1833, 176–183. 10.1016/j.bbamcr.2012.08.004 22902477

[B7] Bernstein H. G. Keilhoff G. Dobrowolny H. Steiner J. (2020). Enhanced mitochondrial autophagy (mitophagy) in oligodendrocytes might play a role in white matter pathology in schizophrenia. Med. Hypotheses 134:109443. 10.1016/j.mehy.2019.109443 31644973

[B8] Brouillet E. Jenkins B. G. Hyman B. T. Ferrante R. J. Kowall N. W. Srivastava R.. (1993). Age-dependent vulnerability of the striatum to the mitochondrial toxin 3-nitropropionic acid. J. Neurochem. 60, 356–359. 10.1111/j.1471-4159.1993.tb05859.x 8417157

[B9] Cai Q. Jeong Y. Y. (2020). Mitophagy in Alzheimer's disease and other age-related neurodegenerative diseases. Cells 9:150. 10.3390/cells9010150 31936292 PMC7017092

[B10] Cai Q. Lu L. Tian J. H. Zhu Y. B. Qiao H. Sheng Z. H. (2010). Snapin-regulated late endosomal transport is critical for efficient autophagy-lysosomal function in neurons. Neuron 68, 73–86. 10.1016/j.neuron.2010.09.022 20920792 PMC2953270

[B11] Cai Q. Zakaria H. M. Sheng Z. H. (2012a). Long time-lapse imaging reveals unique features of PARK2/Parkin-mediated mitophagy in mature cortical neurons. Autophagy 8, 976–978. 10.4161/auto.20218 22739253 PMC3427264

[B12] Cai Q. Zakaria H. M. Simone A. Sheng Z. H. (2012b). Spatial parkin translocation and degradation of damaged mitochondria via mitophagy in live cortical neurons. Curr. Biol. 22, 545–552. 10.1016/j.cub.2012.02.005 22342752 PMC3313683

[B13] Chen Y. Dorn G. W. II. (2013). PINK1-phosphorylated mitofusin 2 is a Parkin receptor for culling damaged mitochondria. Science 340, 471–475. 10.1126/science.1231031 23620051 PMC3774525

[B14] Cherra S. J. III Steer E. Gusdon A. M. Kiselyov K. Chu C. T. (2013). Mutant LRRK2 elicits calcium imbalance and depletion of dendritic mitochondria in neurons. Am. J. Pathol. 182, 474–484. 10.1016/j.ajpath.2012.10.027 23231918 PMC3562730

[B15] Chu C. T. (2019). Multiple pathways for mitophagy: a neurodegenerative conundrum for Parkinson's disease. Neurosci. Lett. 697, 66–71. 10.1016/j.neulet.2018.04.004 29626647 PMC6170746

[B16] Conze D. Brenner C. Kruger C. L. (2019). Safety and metabolism of long-term administration of NIAGEN (Nicotinamide Riboside Chloride) in a randomized, double-blind, placebo-controlled clinical trial of healthy overweight adults. Sci. Rep. 9:9772. 10.1038/s41598-019-46120-z 31278280 PMC6611812

[B17] Cummins N. Tweedie A. Zuryn S. Bertran-Gonzalez J. Gotz J. (2019). Disease-associated tau impairs mitophagy by inhibiting Parkin translocation to mitochondria. EMBO J. 38:e99360. 10.15252/embj.201899360 30538104 PMC6356067

[B18] Dave K. D. De Silva S. Sheth N. P. Ramboz S. Beck M. J. Quang C.. (2014). Phenotypic characterization of recessive gene knockout rat models of Parkinson's disease. Neurobiol. Dis. 70, 190–203. 10.1016/j.nbd.2014.06.009 24969022

[B19] De Brito O. M. Scorrano L. (2008). Mitofusin 2 tethers endoplasmic reticulum to mitochondria. Nature 456, 605–610. 10.1038/nature07534 19052620

[B20] Delettre C. Lenaers G. Griffoin J. M. Gigarel N. Lorenzo C. Belenguer P.. (2000). Nuclear gene OPA1, encoding a mitochondrial dynamin-related protein, is mutated in dominant optic atrophy. Nat. Genet. 26, 207–210. 10.1038/79936 11017079

[B21] Devi T. S. Yumnamcha T. Yao F. Somayajulu M. Kowluru R. A. Singh L. P. (2019). TXNIP mediates high glucose-induced mitophagic flux and lysosome enlargement in human retinal pigment epithelial cells. Biol. Open 8:bio038521. 10.1242/bio.038521 31023645 PMC6503994

[B22] Devireddy S. Liu A. Lampe T. Hollenbeck P. J. (2015). The organization of mitochondrial quality control and life cycle in the nervous system *in vivo* in the absence of PINK1. J. Neurosci. 35, 9391–9401. 10.1523/JNEUROSCI.1198-15.2015 26109662 PMC4478254

[B23] Dollerup O. L. Christensen B. Svart M. Schmidt M. S. Sulek K. Ringgaard S.. (2018). A randomized placebo-controlled clinical trial of nicotinamide riboside in obese men: safety, insulin-sensitivity, and lipid-mobilizing effects. Am. J. Clin. Nutr. 108, 343–353. 10.1093/ajcn/nqy132 29992272

[B24] Eisenberg T. Abdellatif M. Schroeder S. Primessnig U. Stekovic S. Pendl T.. (2016). Cardioprotection and lifespan extension by the natural polyamine spermidine. Nat. Med. 22, 1428–1438. 10.1038/nm.4222 27841876 PMC5806691

[B25] Eisenberg T. Knauer H. Schauer A. Buttner S. Ruckenstuhl C. Carmona-Gutierrez D.. (2009). Induction of autophagy by spermidine promotes longevity. Nat. Cell Biol. 11, 1305–1314. 10.1038/ncb1975 19801973

[B26] Evans C. S. Holzbaur E. L. (2020a). Degradation of engulfed mitochondria is rate-limiting in Optineurin-mediated mitophagy in neurons. Elife 9:e50260. 10.7554/eLife.50260 31934852 PMC6959996

[B27] Evans C. S. Holzbaur E. L. F. (2020b). Quality control in neurons: mitophagy and other selective autophagy mechanisms. J. Mol. Biol. 432, 240–260. 10.1016/j.jmb.2019.06.031 31295455 PMC6946890

[B28] Fang E. F. (2019). Mitophagy and NAD(+) inhibit Alzheimer disease. Autophagy 15, 1112–1114. 10.1080/15548627.2019.1596497 30922179 PMC6526831

[B29] Fang E. F. Hou Y. Palikaras K. Adriaanse B. A. Kerr J. S. Yang B.. (2019). Mitophagy inhibits amyloid-beta and tau pathology and reverses cognitive deficits in models of Alzheimer's disease. Nat. Neurosci. 22, 401–412. 10.1038/s41593-018-0332-9 30742114 PMC6693625

[B30] Filadi R. Greotti E. Turacchio G. Luini A. Pozzan T. Pizzo P. (2015). Mitofusin 2 ablation increases endoplasmic reticulum-mitochondria coupling. Proc. Natl. Acad. Sci. U. S. A. 112, E2174–2181. 10.1073/pnas.1504880112 25870285 PMC4418914

[B31] Franco-Iborra S. Plaza-Zabala A. Montpeyo M. Sebastian D. Vila M. Martinez-Vicente M. (2020). Mutant HTT (huntingtin) impairs mitophagy in a cellular model of Huntington disease. Autophagy. 1–18. 10.1080/15548627.2020.1728096 PMC8032238 32093570

[B32] Fu Y. He F. Zhang S. Jiao X. (1995). Consistent striatal damage in rats induced by 3-nitropropionic acid and cultures of arthrinium fungus. Neurotoxicol. Teratol. 17, 413–418. 10.1016/0892-0362(94)00078-R 7565487

[B33] Garcia G. C. Bartol T. M. Phan S. Bushong E. A. Perkins G. Sejnowski T. J.. (2019). Mitochondrial morphology provides a mechanism for energy buffering at synapses. Sci. Rep. 9:18306. 10.1038/s41598-019-54159-1 31797946 PMC6893035

[B34] Garcia-Prat L. Sousa-Victor P. Munoz-Canoves P. (2017). Proteostatic and metabolic control of stemness. Cell Stem Cell 20, 593–608. 10.1016/j.stem.2017.04.011 28475885

[B35] Gatica D. Lahiri V. Klionsky D. J. (2018). Cargo recognition and degradation by selective autophagy. Nat. Cell Biol. 20, 233–242. 10.1038/s41556-018-0037-z 29476151 PMC6028034

[B36] Georgakopoulos N. D. Wells G. Campanella M. (2017). The pharmacological regulation of cellular mitophagy. Nat. Chem. Biol. 13, 136–146. 10.1038/nchembio.2287 28103219

[B37] Gilmour B. C. Gudmundsrud R. Frank J. Hov A. Lautrup S. Aman Y.. (2020). Targeting NAD(+) in translational research to relieve diseases and conditions of metabolic stress and ageing. Mech. Ageing Dev. 186:111208. 10.1016/j.mad.2020.111208 31953124

[B38] Goldberg M. S. Fleming S. M. Palacino J. J. Cepeda C. Lam H. A. Bhatnagar A.. (2003). Parkin-deficient mice exhibit nigrostriatal deficits but not loss of dopaminergic neurons. J. Biol. Chem. 278, 43628–43635. 10.1074/jbc.M308947200 12930822

[B39] Guo X. Sun X. Hu D. Wang Y. J. Fujioka H. Vyas R.. (2016). VCP recruitment to mitochondria causes mitophagy impairment and neurodegeneration in models of Huntington's disease. Nat. Commun. 7:12646. 10.1038/ncomms12646 27561680 PMC5007466

[B40] Gupta V. K. Pech U. Bhukel A. Fulterer A. Ender A. Mauermann S. F.. (2016). Spermidine suppresses age-associated memory impairment by preventing adverse increase of presynaptic active zone size and release. PLoS Biol. 14:e1002563. 10.1371/journal.pbio.1002563 27684064 PMC5042543

[B41] Gupta V. K. Scheunemann L. Eisenberg T. Mertel S. Bhukel A. Koemans T. S.. (2013). Restoring polyamines protects from age-induced memory impairment in an autophagy-dependent manner. Nat. Neurosci. 16, 1453–1460. 10.1038/nn.3512 23995066

[B42] Guyot M. C. Hantraye P. Dolan R. Palfi S. Maziere M. Brouillet E. (1997). Quantifiable bradykinesia, gait abnormalities and Huntington's disease-like striatal lesions in rats chronically treated with 3-nitropropionic acid. Neuroscience 79, 45–56. 10.1016/S0306-4522(96)00602-1 9178864

[B43] Han S. Jeong Y. Y. Sheshadri P. Su X. Cai Q. (2020). Mitophagy regulates integrity of mitochondria at synapses and is critical for synaptic maintenance. EMBO Rep. 21:e49801. 10.15252/embr.201949801 32627320 PMC7507095

[B44] Harper J. W. Ordureau A. Heo J. M. (2018). Building and decoding ubiquitin chains for mitophagy. Nat. Rev. Mol. Cell Biol. 19, 93–108. 10.1038/nrm.2017.129 29358684

[B45] Hasson S. A. Kane L. A. Yamano K. Huang C. H. Sliter D. A. Buehler E.. (2013). High-content genome-wide RNAi screens identify regulators of parkin upstream of mitophagy. Nature 504, 291–295. 10.1038/nature12748 24270810 PMC5841086

[B46] He F. Zhang S. Qian F. Zhang C. (1995). Delayed dystonia with striatal CT lucencies induced by a mycotoxin (3-nitropropionic acid). Neurology 45, 2178–2183. 10.1212/WNL.45.12.2178 8848189

[B47] Heo J. M. Ordureau A. Paulo J. A. Rinehart J. Harper J. W. (2015). The PINK1-PARKIN mitochondrial ubiquitylation pathway drives a program of OPTN/NDP52 recruitment and TBK1 activation to promote mitophagy. Mol. Cell 60, 7–20. 10.1016/j.molcel.2015.08.016 26365381 PMC4592482

[B48] Hipp M. S. Kasturi P. Hartl F. U. (2019). The proteostasis network and its decline in ageing. Nat. Rev. Mol. Cell Biol. 20, 421–435. 10.1038/s41580-019-0101-y 30733602

[B49] Holtzman E. Novikoff A. B. (1965). Lysomes in the rat sciatic nerve following crush. J. Cell Biol. 27, 651–669. 10.1083/jcb.27.3.651 5885432 PMC2106775

[B50] Hou Y. Dan X. Babbar M. Wei Y. Hasselbalch S. G. Croteau D. L.. (2019). Ageing as a risk factor for neurodegenerative disease. Nat. Rev. Neurol. 15, 565–581. 10.1038/s41582-019-0244-7 31501588

[B51] Hou Y. Lautrup S. Cordonnier S. Wang Y. Croteau D. L. Zavala E.. (2018). NAD(+) supplementation normalizes key Alzheimer's features and DNA damage responses in a new AD mouse model with introduced DNA repair deficiency. Proc. Natl. Acad. Sci. U. S. A. 115, E1876–E1885. 10.1073/pnas.1718819115 29432159 PMC5828618

[B52] Hwang S. Disatnik M. H. Mochly-Rosen D. (2015). Impaired GAPDH-induced mitophagy contributes to the pathology of Huntington's disease. EMBO Mol. Med. 7, 1307–1326. 10.15252/emmm.201505256 26268247 PMC4604685

[B53] Joaquim H. P. G. Costa A. C. Forlenza O. V. Gattaz W. F. Talib L. L. (2019). Decreased plasmatic spermidine and increased spermine in mild cognitive impairment and Alzheimer's disease patients. Arch. Clin. Psych. 46, 120–124. 10.1590/0101-60830000000209

[B54] Kerr J. S. Adriaanse B. A. Greig N. H. Mattson M. P. Cader M. Z. Bohr V. A. (2017). Mitophagy and Alzheimer's disease: cellular and molecular mechanisms. Trends Neurosci. 40, 151–166. 10.1016/j.tins.2017.01.002 28190529 PMC5341618

[B55] Khacho M. Harris R. Slack R. S. (2019). Mitochondria as central regulators of neural stem cell fate and cognitive function. Nat. Rev. Neurosci. 20, 34–48. 10.1038/s41583-018-0091-3 30464208

[B56] Khalil B. El Fissi N. Aouane A. Cabirol-Pol M. J. Rival T. Lievens J. C. (2015). PINK1-induced mitophagy promotes neuroprotection in Huntington's disease. Cell Death Dis. 6:e1617. 10.1038/cddis.2014.581 25611391 PMC4669776

[B57] Khaminets A. Behl C. Dikic I. (2016). Ubiquitin-dependent and independent signals in selective autophagy. Trends Cell Biol. 26, 6–16. 10.1016/j.tcb.2015.08.010 26437584

[B58] Kitada T. Asakawa S. Hattori N. Matsumine H. Yamamura Y. Minoshima S.. (1998). Mutations in the parkin gene cause autosomal recessive juvenile parkinsonism. Nature 392, 605–608. 10.1038/33416 9560156

[B59] Lautrup S. Sinclair D. A. Mattson M. P. Fang E. F. (2019). NAD(+) in brain aging and neurodegenerative disorders. Cell Metab. 30, 630–655. 10.1016/j.cmet.2019.09.001 31577933 PMC6787556

[B60] Lazarou M. Sliter D. A. Kane L. A. Sarraf S. A. Wang C. Burman J. L.. (2015). The ubiquitin kinase PINK1 recruits autophagy receptors to induce mitophagy. Nature 524, 309–314. 10.1038/nature14893 26266977 PMC5018156

[B61] Lee J. J. Sanchez-Martinez A. Zarate A. M. Beninca C. Mayor U. Clague M. J. (2018). Basal mitophagy is widespread in Drosophila but minimally affected by loss of Pink1 or parkin. J. Cell Biol. 217, 1613–1622. 10.1083/jcb.201801044 29500189 PMC5940313

[B62] Lichtenwalner R. J. Forbes M. E. Bennett S. A. Lynch C. D. Sonntag W. E. Riddle D. R. (2001). Intracerebroventricular infusion of insulin-like growth factor-I ameliorates the age-related decline in hippocampal neurogenesis. Neuroscience 107, 603–613. 10.1016/S0306-4522(01)00378-5 11720784

[B63] Lie P. P. Y. Nixon R. A. (2019). Lysosome trafficking and signaling in health and neurodegenerative diseases. Neurobiol. Dis. 122, 94–105. 10.1016/j.nbd.2018.05.015 29859318 PMC6381838

[B64] Lin M. Y. Cheng X. T. Tammineni P. Xie Y. Zhou B. Cai Q.. (2017). Releasing syntaphilin removes stressed mitochondria from axons independent of mitophagy under pathophysiological conditions. Neuron 94, 595–610. 10.1016/j.neuron.2017.04.004 28472658 PMC5484086

[B65] Liu J. Liu W. Li R. Yang H. (2019). Mitophagy in Parkinson's disease: from pathogenesis to treatment. Cells 8:712. 10.3390/cells8070712 31336937 PMC6678174

[B66] Lou G. Palikaras K. Lautrup S. Scheibye-Knudsen M. Tavernarakis N. Fang E. F. (2020). Mitophagy and neuroprotection. Trends Mol. Med. 26, 8–20. 10.1016/j.molmed.2019.07.002 31375365

[B67] Madeo F. Eisenberg T. Pietrocola F. Kroemer G. (2018). Spermidine in health and disease. Science 359:eaan2788. 10.1126/science.aan2788 29371440

[B68] Maglione M. Kochlamazashvili G. Eisenberg T. Racz B. Michael E. Toppe D.. (2019). Spermidine protects from age-related synaptic alterations at hippocampal mossy fiber-CA3 synapses. Sci. Rep. 9:19616. 10.1038/s41598-019-56133-3 31873156 PMC6927957

[B69] Martinez-Vicente M. Talloczy Z. Wong E. Tang G. Koga H. Kaushik S.. (2010). Cargo recognition failure is responsible for inefficient autophagy in Huntington's disease. Nat. Neurosci. 13, 567–576. 10.1038/nn.2528 20383138 PMC2860687

[B70] Martin-Maestro P. Gargini R. Perry G. Avila J. Garcia-Escudero V. (2016). PARK2 enhancement is able to compensate mitophagy alterations found in sporadic Alzheimer's disease. Hum. Mol. Genet. 25, 792–806. 10.1093/hmg/ddv616 26721933 PMC4743695

[B71] Mclelland G. L. Goiran T. Yi W. Dorval G. Chen C. X. Lauinger N. D.. (2018). Mfn2 ubiquitination by PINK1/parkin gates the p97-dependent release of ER from mitochondria to drive mitophagy. Elife 7:32. 10.7554/eLife.32866.032 29676259 PMC5927771

[B72] Mcwilliams T. G. Prescott A. R. Allen G. F. Tamjar J. Munson M. J. Thomson C.. (2016). mito-QC illuminates mitophagy and mitochondrial architecture *in vivo*. J. Cell Biol. 214, 333–345. 10.1083/jcb.201603039 27458135 PMC4970326

[B73] Mcwilliams T. G. Prescott A. R. Montava-Garriga L. Ball G. Singh F. Barini E.. (2018). Basal mitophagy occurs independently of PINK1 in mouse tissues of high metabolic demand. Cell Metab. 27, 439–449. 10.1016/j.cmet.2017.12.008 29337137 PMC5807059

[B74] Misgeld T. Schwarz T. L. (2017). Mitostasis in neurons: maintaining mitochondria in an extended cellular architecture. Neuron 96, 651–666. 10.1016/j.neuron.2017.09.055 29096078 PMC5687842

[B75] Misko A. L. Sasaki Y. Tuck E. Milbrandt J. Baloh R. H. (2012). Mitofusin2 mutations disrupt axonal mitochondrial positioning and promote axon degeneration. J. Neurosci. 32, 4145–4155. 10.1523/JNEUROSCI.6338-11.2012 22442078 PMC3319368

[B76] Montava-Garriga L. Ganley I. G. (2020). Outstanding questions in mitophagy: what we do and do not know. J. Mol. Biol. 432, 206–230. 10.1016/j.jmb.2019.06.032 31299243

[B77] Ordureau A. Paulo J. A. Zhang J. An H. Swatek K. N. Cannon J. R.. (2020). Global landscape and dynamics of parkin and USP30-dependent ubiquitylomes in ineurons during mitophagic signaling. Mol. Cell 77, 1124–1142. 10.1016/j.molcel.2019.11.013 32142685 PMC7098486

[B78] Ordureau A. Paulo J. A. Zhang W. Ahfeldt T. Zhang J. Cohn E. F.. (2018). Dynamics of PARKIN-dependent mitochondrial ubiquitylation in induced neurons and model systems revealed by digital snapshot proteomics. Mol. Cell 70, 211–227. 10.1016/j.molcel.2018.03.012 29656925 PMC5910199

[B79] Orr A. L. Li S. Wang C. E. Li H. Wang J. Rong J.. (2008). N-terminal mutant huntingtin associates with mitochondria and impairs mitochondrial trafficking. J. Neurosci. 28, 2783–2792. 10.1523/JNEUROSCI.0106-08.2008 18337408 PMC2652473

[B80] Palikaras K. Lionaki E. Tavernarakis N. (2015). Coordination of mitophagy and mitochondrial biogenesis during ageing in *C. elegans*. Nature 521, 525–528. 10.1038/nature14300 25896323

[B81] Palikaras K. Lionaki E. Tavernarakis N. (2018). Mechanisms of mitophagy in cellular homeostasis, physiology and pathology. Nat. Cell Biol. 20, 1013–1022. 10.1038/s41556-018-0176-2 30154567

[B82] Palikaras K. Tavernarakis N. (2014). Mitochondrial homeostasis: the interplay between mitophagy and mitochondrial biogenesis. Exp. Gerontol. 56, 182–188. 10.1016/j.exger.2014.01.021 24486129

[B83] Palikaras K. Tavernarakis N. (2020). Regulation and roles of mitophagy at synapses. Mech. Ageing Dev. 187:111216. 10.1016/j.mad.2020.111216 32084458

[B84] Park H. Chung K. M. An H. K. Gim J. E. Hong J. Woo H.. (2019). Parkin promotes mitophagic cell death in adult hippocampal neural stem cells following insulin withdrawal. Front. Mol. Neurosci. 12:46. 10.3389/fnmol.2019.00046 30853892 PMC6395409

[B85] Perez F. A. Palmiter R. D. (2005). Parkin-deficient mice are not a robust model of parkinsonism. Proc. Natl. Acad. Sci. U. S. A. 102, 2174–2179. 10.1073/pnas.0409598102 15684050 PMC548311

[B86] Pickford F. Masliah E. Britschgi M. Lucin K. Narasimhan R. Jaeger P. A.. (2008). The autophagy-related protein beclin 1 shows reduced expression in early Alzheimer disease and regulates amyloid beta accumulation in mice. J. Clin. Invest. 118, 2190–2199. 10.1172/JCI33585 18497889 PMC2391284

[B87] Pickles S. Vigie P. Youle R. J. (2018). Mitophagy and quality control mechanisms in mitochondrial maintenance. Curr. Biol. 28, 170–185. 10.1016/j.cub.2018.01.004 29462587 PMC7255410

[B88] Pickrell A. M. Youle R. J. (2015). The roles of PINK1, parkin, and mitochondrial fidelity in Parkinson's disease. Neuron 85, 257–273. 10.1016/j.neuron.2014.12.007 25611507 PMC4764997

[B89] Puri R. Cheng X. T. Lin M. Y. Huang N. Sheng Z. H. (2019). Mul1 restrains Parkin-mediated mitophagy in mature neurons by maintaining ER-mitochondrial contacts. Nat. Commun. 10:3645. 10.1038/s41467-019-11636-5 31409786 PMC6692330

[B90] Radenkovic D. Reason Verdin E. (2020). Clinical evidence for targeting NAD therapeutically. Pharmaceuticals 13:247. 10.3390/ph13090247 32942582 PMC7558103

[B91] Rawson R. L. Yam L. Weimer R. M. Bend E. G. Hartwieg E. Horvitz H. R.. (2014). Axons degenerate in the absence of mitochondria in *C. elegans*. Curr. Biol. 24, 760–765. 10.1016/j.cub.2014.02.025 24631238 PMC4018749

[B92] Schondorf D. C. Ivanyuk D. Baden P. Sanchez-Martinez A. De Cicco S. Yu C.. (2018). The NAD+ precursor nicotinamide riboside rescues mitochondrial defects and neuronal loss in iPSC and fly models of Parkinson's disease. Cell Rep. 23, 2976–2988. 10.1016/j.celrep.2018.05.009 29874584

[B93] Sekine S. Youle R. J. (2018). PINK1 import regulation; a fine system to convey mitochondrial stress to the cytosol. BMC Biol. 16:2. 10.1186/s12915-017-0470-7 29325568 PMC5795276

[B94] Sentelle R. D. Senkal C. E. Jiang W. Ponnusamy S. Gencer S. Selvam S. P.. (2012). Ceramide targets autophagosomes to mitochondria and induces lethal mitophagy. Nat. Chem. Biol. 8, 831–838. 10.1038/nchembio.1059 22922758 PMC3689583

[B95] Sharma M. Jarquin U. N. R. Rivera O. Kazantzis M. Eshraghi M. Shahani N.. (2019). Rhes, a striatal-enriched protein, promotes mitophagy via Nix. Proc. Natl. Acad. Sci. U. S. A. 116, 23760–23771. 10.1073/pnas.1912868116 31676548 PMC6876193

[B96] Sheng Z. H. Cai Q. (2012). Mitochondrial transport in neurons: impact on synaptic homeostasis and neurodegeneration. Nat. Rev. Neurosci. 13, 77–93. 10.1038/nrn3156 22218207 PMC4962561

[B97] Shi R. Y. Zhu S. H. Li V. Gibson S. B. Xu X. S. Kong J. M. (2014). BNIP3 interacting with LC3 triggers excessive mitophagy in delayed neuronal death in stroke. CNS Neurosci. Ther. 20, 1045–1055. 10.1111/cns.12325 25230377 PMC6492992

[B98] Shirendeb U. Reddy A. P. Manczak M. Calkins M. J. Mao P. Tagle D. A.. (2011). Abnormal mitochondrial dynamics, mitochondrial loss and mutant huntingtin oligomers in Huntington's disease: implications for selective neuronal damage. Hum. Mol. Genet. 20, 1438–1455. 10.1093/hmg/ddr024 21257639 PMC3049363

[B99] Sliter D. A. Martinez J. Hao L. Chen X. Sun N. Fischer T. D.. (2018). Parkin and PINK1 mitigate STING-induced inflammation. Nature 561, 258–262. 10.1038/s41586-018-0448-9 30135585 PMC7362342

[B100] Smith H. L. Bourne J. N. Cao G. Chirillo M. A. Ostroff L. E. Watson D. J.. (2016). Mitochondrial support of persistent presynaptic vesicle mobilization with age-dependent synaptic growth after LTP. Elife 5:14. 10.7554/eLife.15275.014 27991850 PMC5235352

[B101] Somredngan S. Thong-Asa W. (2018). Neurological changes in vulnerable brain areas of chronic cerebral hypoperfusion mice. Ann. Neurosci. 24, 233–242. 10.1159/000481789 29849447 PMC5969357

[B102] Steketee M. B. Moysidis S. N. Weinstein J. E. Kreymerman A. Silva J. P. Iqbal S.. (2012). Mitochondrial dynamics regulate growth cone motility, guidance, and neurite growth rate in perinatal retinal ganglion cells *in vitro*. Invest. Ophthalmol. Vis. Sci. 53, 7402–7411. 10.1167/iovs.12-10298 23049086 PMC3484733

[B103] Su S. H. Wu Y. F. Wang D. P. Hai J. (2018). Inhibition of excessive autophagy and mitophagy mediates neuroprotective effects of URB597 against chronic cerebral hypoperfusion. Cell Death Dis. 9:733. 10.1038/s41419-018-0755-y 29955058 PMC6023888

[B104] Subramaniam S. (2020). Exaggerated mitophagy: a weapon of striatal destruction in the brain? Biochem. Soc. Trans. 48, 709–717. 10.1042/BST20191283 32129826 PMC7200642

[B105] Sun N. Yun J. Liu J. Malide D. Liu C. Rovira I Holmstrom K. M.. (2015). Measuring *in vivo* mitophagy. Mol. Cell 60, 685–696. 10.1016/j.molcel.2015.10.009 26549682 PMC4656081

[B106] Sung H. Tandarich L. C. Nguyen K. Hollenbeck P. J. (2016). Compartmentalized regulation of Parkin-mediated mitochondrial quality control in the drosophila nervous system *in vivo*. J. Neurosci. 36, 7375–7391. 10.1523/JNEUROSCI.0633-16.2016 27413149 PMC4945662

[B107] Verreet T. Weaver C. J. Hino H. Hibi M. Poulain F. E. (2019). Syntaphilin-mediated docking of mitochondria at the growth cone is dispensable for axon elongation *in vivo*. eNeuro 6:ENEURO.0026-19.2019. 10.1523/ENEURO.0026-19.2019 31481398 PMC6751374

[B108] Whitworth A. J. Theodore D. A. Greene J. C. Benes H. Wes P. D. Pallanck L. J. (2005). Increased glutathione S-transferase activity rescues dopaminergic neuron loss in a Drosophila model of Parkinson's disease. Proc. Natl. Acad. Sci. U. S. A. 102, 8024–8029. 10.1073/pnas.0501078102 15911761 PMC1142368

[B109] Wirth M. Benson G. Schwarz C. Kobe T. Grittner U. Schmitz D.. (2018). The effect of spermidine on memory performance in older adults at risk for dementia: a randomized controlled trial. Cortex 109, 181–188. 10.1016/j.cortex.2018.09.014 30388439

[B110] Xie C. Aman Y. Adriaanse B. A. Cader M. Z. Plun-Favreau H. Xiao J. (2019). Culprit or bystander: defective mitophagy in Alzheimer's disease. Front. Cell Dev. Biol. 7:391 10.3389/fcell.2019.00391 32010698 PMC6978796

[B111] Yan X. Wang B. Hu Y. Wang S. Zhang X. (2020). Abnormal mitochondrial quality control in neurodegenerative diseases. Front. Cell. Neurosci. 14:138. 10.3389/fncel.2020.00138 32655368 PMC7324542

[B112] Yang Y. Gehrke S. Imai Y. Huang Z. Ouyang Y. Wang J. W.. (2006). Mitochondrial pathology and muscle and dopaminergic neuron degeneration caused by inactivation of Drosophila Pink1 is rescued by Parkin. Proc. Natl. Acad. Sci. U. S. A. 103, 10793–10798. 10.1073/pnas.0602493103 16818890 PMC1502310

[B113] Yu-Wai-Man P. Griffiths P. G. Gorman G. S. Lourenco C. M. Wright A. F. Auer-Grumbach M.. (2010). Multi-system neurological disease is common in patients with OPA1 mutations. Brain 133, 771–786. 10.1093/brain/awq007 20157015 PMC2842512

[B114] Zaninello M. Palikaras K. Naon D. Iwata K. Herkenne S. Quintana-Cabrera R.. (2020). Inhibition of autophagy curtails visual loss in a model of autosomal dominant optic atrophy. Nat. Commun. 11:4029. 10.1038/s41467-020-17821-1 32788597 PMC7423926

[B115] Zhao T. Hao Y. Kaplan J. M. (2018). Axonal mitochondria modulate neuropeptide secretion through the hypoxic stress response in *Caenorhabditis elegans*. Genetics 210, 275–285. 10.1534/genetics.118.301014 30049781 PMC6116974

